# FV-162 is a novel, orally bioavailable, irreversible proteasome inhibitor with improved pharmacokinetics displaying preclinical efficacy with continuous daily dosing

**DOI:** 10.1038/cddis.2015.187

**Published:** 2015-07-09

**Authors:** Z Wang, P Dove, X Wang, A Shamas-Din, Z Li, A Nachman, Y J Oh, R Hurren, A Ruschak, S Climie, B Press, C Griffin, E Undzys, A Aman, R Al-awar, L E Kay, D O'Neill, S Trudel, M Slassi, A D Schimmer

**Affiliations:** 1 Princess Margaret Cancer Centre, Toronto, ON, Canada; 2 Fluorinov Pharma Inc., Toronto, ON, Canada; 3 Department of Molecular Genetics, Biochemistry and Chemistry, University of Toronto, Toronto, ON, Canada; 4 Ontario Institute for Cancer Research, Toronto, ON, Canada

## Abstract

Approved proteasome inhibitors have advanced the treatment of multiple myeloma but are associated with serious toxicities, poor pharmacokinetics, and most with the inconvenience of intravenous administration. We therefore sought to identify novel orally bioavailable proteasome inhibitors with a continuous daily dosing schedule and improved therapeutic window using a unique drug discovery platform. We employed a fluorine-based medicinal chemistry technology to synthesize 14 novel analogs of epoxyketone-based proteasome inhibitors and screened them for their stability, ability to inhibit the chymotrypsin-like proteasome, and antimyeloma activity *in vitro*. The tolerability, pharmacokinetics, pharmacodynamic activity, and antimyeloma efficacy of our lead candidate were examined in NOD/SCID mice. We identified a tripeptide epoxyketone, FV-162, as a metabolically stable, potent proteasome inhibitor cytotoxic to human myeloma cell lines and primary myeloma cells. FV-162 had limited toxicity and was well tolerated on a continuous daily dosing schedule. Compared with the benchmark oral irreversible proteasome inhibitor, ONX-0192, FV-162 had a lower peak plasma concentration and longer half-life, resulting in a larger area under the curve (AUC). Oral FV-162 treatment induced rapid, irreversible inhibition of chymotrypsin-like proteasome activity in murine red blood cells and inhibited tumor growth in a myeloma xenograft model. Our data suggest that oral FV-162 with continuous daily dosing schedule displays a favorable safety, efficacy, and pharmacokinetic profile *in vivo*, identifying it as a promising lead for clinical evaluation in myeloma therapy.

The ubiquitin–proteasome system is responsible for the regulation and degradation of the majority of the intracellular proteins in eukaryotic cells.^1^ The 26S proteasome is a multi-subunit protein complex that mediates the proteolytic degradation and turnover of damaged, misfolded or excess proteins that have been polyubiquitylated in the cytoplasm and nucleus.^1, 2^ The 26S proteasome consists of a 20S core particle, capped by 19S regulatory particles.^3, 4^ The 19S particle participates in the recognition, processing, unfolding, and translocation of ubiquitylated protein substrates into the 20S core.^5^ Substrates are then degraded inside the chamber of the barrel-like 20S core particle, where the active sites of multiple *β*1, *β*2, and *β*5 subunits catalyze caspase-like (C-L), trypsin-like (T-L), or chymotrypsin-like (CT-L) proteolysis, respectively.^6, 7^ Inhibition of the 26S proteasome activity leads to disruption of the cell cycle and induction of apoptosis.^8^

Cancer cells have an increased dependency on the integrity of the ubiquitin–proteasome system machinery compared with normal cells in preclinical studies. This finding is predominantly evident in hematological malignancies, identifying the 26S proteasome as a promising anticancer therapeutic target.^9, 10, 11, 12^ In particular, cells derived from multiple myeloma are notably sensitive to proteasome inhibition, at least in part, owing to their characteristically high rates of immunoglobulin protein biosynthesis and increased proteasome activity.^13, 14, 15^ The continuous activity of the proteasome in myeloma cells makes them particularly susceptible to prolonged inhibition.^16^

Bortezomib, the first proteasome inhibitor approved for clinical use, is a dipeptide boronic acid that reversibly binds to the active site of the *β*5 and *β*1 subunit to competitively inhibit proteasome function.^9, 10, 17^ By inhibiting the proteasome, bortezomib acts through multiple cellular pathways that ultimately result in cell cycle arrest and apoptosis.^18^ Bortezomib is currently approved for the treatment of newly diagnosed, relapsed or refractory multiple myeloma and mantle cell lymphoma.^18^ Carfilzomib was subsequently developed as a second-generation inhibitor that belongs to the epoxyketone class and irreversibly binds to the active site of the *β*5 subunit of the proteasome. Carfilzomib is structurally and mechanistically distinct from bortezomib and overcomes bortezomib resistance in multiple myeloma cell lines and in primary multiple myeloma cells from patients.^17, 19^ Carfilzomib is currently also approved for relapsed and refractory multiple myeloma. ONX-0912 (also known as oprozomib) is another epoxyketone class oral proteasome inhibitor that is an analog of carfilzomib.^20, 21^ Similar to carfilzomib, ONX-0912 promotes cell death in primary myeloma cells from patients who relapsed after treatment with bortezomib.^20^ ONX-0912 has advanced into phase I/II trials in hematological and solid malignancies.^22, 23, 24^

Despite their clinical efficacy, treatment with proteasome inhibitors is associated with a number of toxicities, including neuropathy, thrombocytopenia, and cardiotoxicity.^25, 26, 27^ The toxicity of currently available proteasome inhibitors necessitates administering the drugs in intermittent dosing schedules, typically biweekly. Although intermittent dosing permits proteasome activity in normal tissues during dose holidays, it has been shown to be sub-optimal for therapy in malignant cells.^16^ Moreover, infrequent administration at relatively high exposures may give rise to undesirable and potentially unnecessary toxicities in normal cells. Potentially, by moderating exposures, an optimized oral proteasome inhibitor with continuous daily dosing could be developed that exploits the high proteasome dependency in malignant cells while sparing normal cells.

In the present study, we report the development of FV-162, a novel, metabolically stable and orally bioavailable epoxyketone-based proteasome inhibitor. FV-162 displays potent anticancer activity and maintains a wide differential activity between malignant and normal cells despite a continuous daily dosing schedule in multiple myeloma cell lines, primary patient cells, and animal models. Overall, our results show that FV-162 inhibits the proteasome, displays metabolic stability, and has a favorable toxicity profile.

## Results

### Synthesis and identification of a novel proteasome inhibitor, FV-162

**Figure 1:**
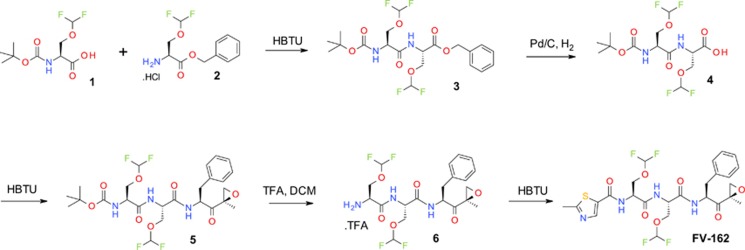
Synthesis of FV-162. FV-162 is a novel structural analog of the irreversible epoxyketone proteasome inhibitors carfilzomib and ONX-0912. It was synthesized in this study using a novel fluorine-based chemistry technology (Fluorinov Pharma Inc., Toronto, ON, Canada). Reaction details and characterizations are given in Materials and Methods

To identify novel orally bioavailable proteasome inhibitors with improved pharmacokinetics and toxicity, we applied a unique fluorine-based medicinal chemistry platform to design and synthesize a diverse set of tripeptide and tetrapeptide epoxyketones, using the structures of ONX-0912 and carfilzomib as chemical backbones. The incorporation of difluoromethoxy moieties into the chemical scaffolds was aimed to improve metabolic stability, decrease toxicity, and increase oral bioavailability by increasing drug exposure (area under the curve (AUC)), whereas lowering the peak plasma concentration (*C*_max_) and prolonging half-life *t*_1/2_.^28^ Of the 14 unique structural analogs evaluated (Supplementary Figure S1), FV-162 was the lead compound based on potency in proteasome inhibition and cell viability assays (Supplementary Figure S2). Therefore, we focused on FV-162 and compared its activity with the orally bioavailable irreversible proteasome inhibitor, ONX-0912. The methodology of synthesis and the structure of FV-162 are outlined in Figure 1.

### FV-162 is a metabolically stable proteasome inhibitor with potent antimyeloma activity

**Figure 2:**
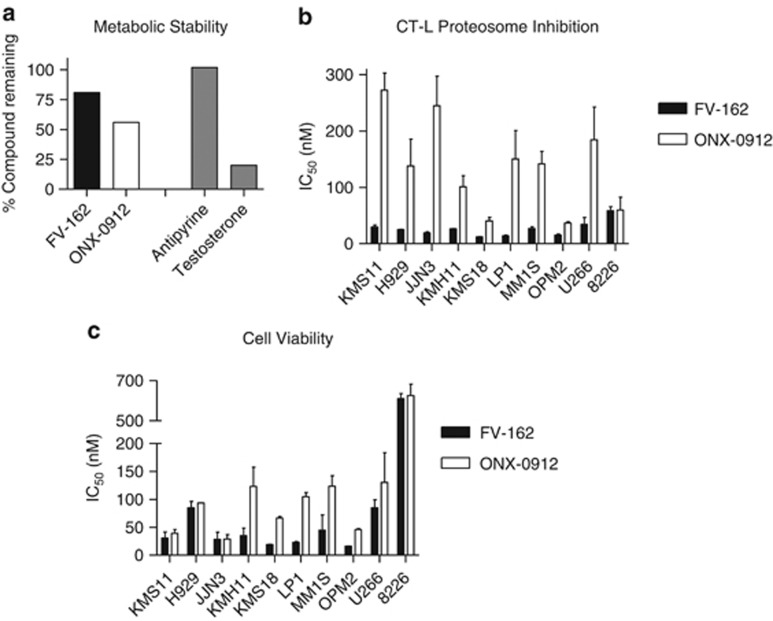
FV-162 is a novel proteasome inhibitor with potent antimyeloma activity. (**a**) The metabolic stability of the compounds shown was determined in the presence of pooled mouse liver microsomes. The percentage of 5 *μ*M of each compound remaining after an incubation of 15 min with 0.5 mg/ml liver microsomes was detected by LC/MS. Antipyrine and testosterone (5 *μ*M each) were included as positive and negative controls, respectively (gray bars). One representative experiment is shown. (**b**) CT-L proteasome activity present in indicated whole cell lysates of 10 human myeloma cell lines was determined through specific cleavage of the fluorogenic substrate Suc-Leu-Leu-Val-Tyr-AMC following exposure to each compound for 2 h. Sensitivity was evaluated using the half-maximal inhibitory concentration (IC_50_). (mean±S.D., *n*=3). (**c**) Cell growth and viability of 10 human myeloma cell lines was assessed using the MTS assay after exposure to each compound for 72 h. Sensitivity was evaluated using the IC_50_. (mean±S.D. *n*=3)

To assess the metabolic stability of FV-162, we incubated the compound with mouse liver microsomes. The percentage of parent compound remaining after 15 min was measured by liquid chromatography/mass spectroscopy (LC/MS) as an indicator of metabolic stability. Over 80% of FV-162 remained intact after incubation with liver microsomes. This was similar to the stability of carfilzomib at 70% (Supplementary Figure S2A) and higher than the stability of ONX-0912 at 56% (Figure 2a).

Consistent with its design as a proteasome inhibitor, FV-162 inhibited the enzymatic activity of the CT-L proteasome subunit when added to total cell lysates from 10 different human myeloma cells. The IC_50_ values of FV-162 were lower than 60 nM for all the cell lines tested. In 6 of the 10 cell lines, FV-162 was over five times more potent than ONX-0912 (Figure 2b). Consistent with its ability to inhibit the proteasome, FV-162 decreased cell growth and viability in all the 10 human myeloma cell lines as measured by 3-(4,5-dimethylthiazol-2-yl)-5-(3-carboxymethoxyphenyl)-2-(4-sulfophenyl)-2H-tetrazolium (MTS) assay. In 5 of the 10 cell lines, FV-162 was over two times more potent than ONX-0912 (Figure 2c). Cell death was independently confirmed by trypan blue staining (data not shown).

**Figure 3:**
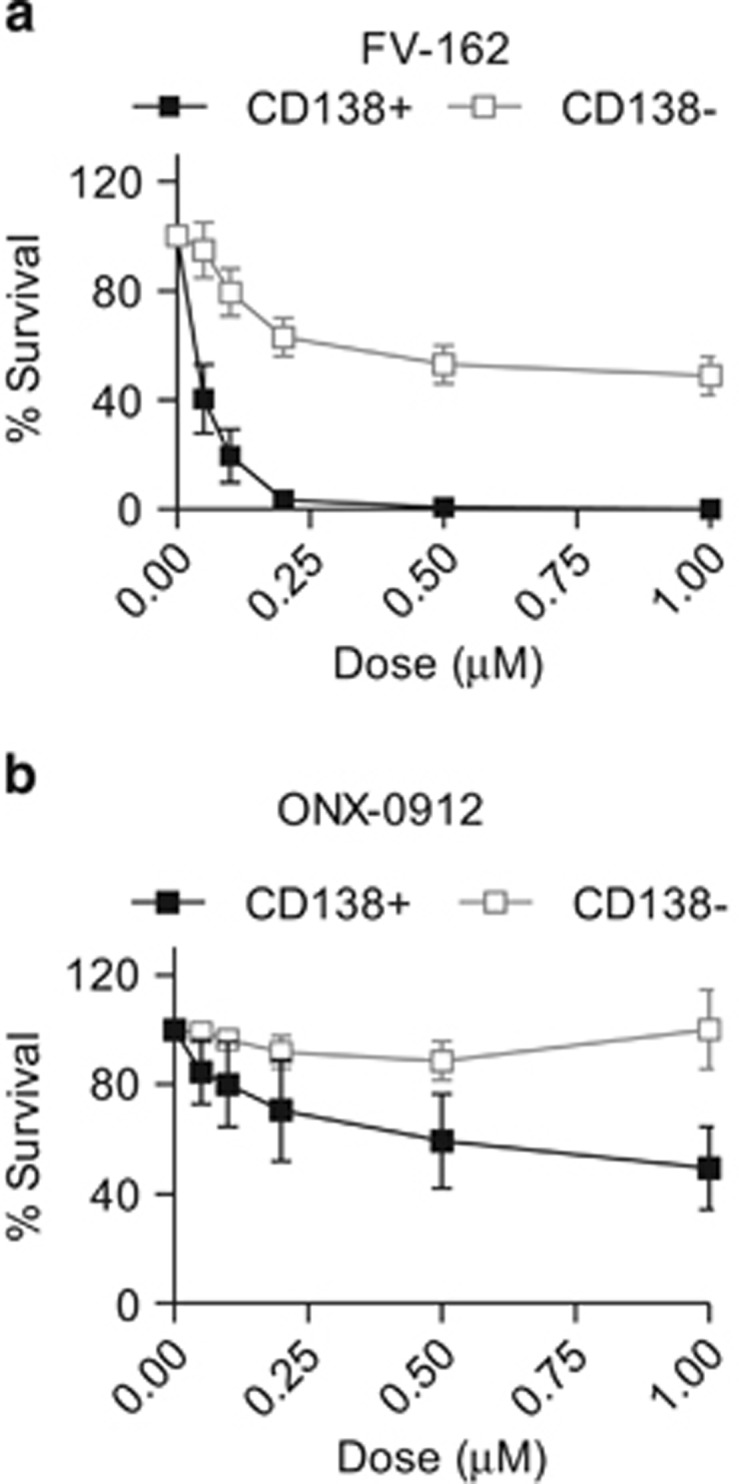
FV-162 selectively induces cell death in primary human myeloma cells. The effects of (**a**) FV-162 and (**b**) ONX-0912 on the viability of primary mononuclear cells isolated from the bone marrow of patients with multiple myeloma. Survival was calculated by detecting Annexin V staining and flow cytometry following 48 h of treatment, relative to vehicle control. FV-162 selectively reduced the viability of primary CD138^+^ myeloma cells over CD138^−^ normal hematopoietic cells. (mean±S.E.M., *n*=8)

Finally, we examined the preclinical efficacy of FV-162 in primary myeloma patient samples. Mononuclear cells isolated from the bone marrow of eight multiple myeloma patients were treated with FV-162 or ONX-0912 for 48 h. After treatment, the percentage of viable myeloma (CD138^+^) and normal hematopoietic (CD138^−^) cells was measured by flow cytometry by staining with Annexin V. At least 80% of primary myeloma cells were killed by FV-162 at concentrations ⩽0.1 *μ*M while normal hematopoietic cells were largely insensitive (Figure 3a). At a five-fold higher concentration of 0.5 *μ*M, FV-162 was toxic to 50% of CD138^−^ cells. In contrast, similar concentrations of ONX-0912 were less effective at inducing death in primary myeloma cells but had no adverse effects on CD138^−^ cells (Figure 3b).

### Pharmacokinetics of FV-162 in rodents

**Figure 4:**
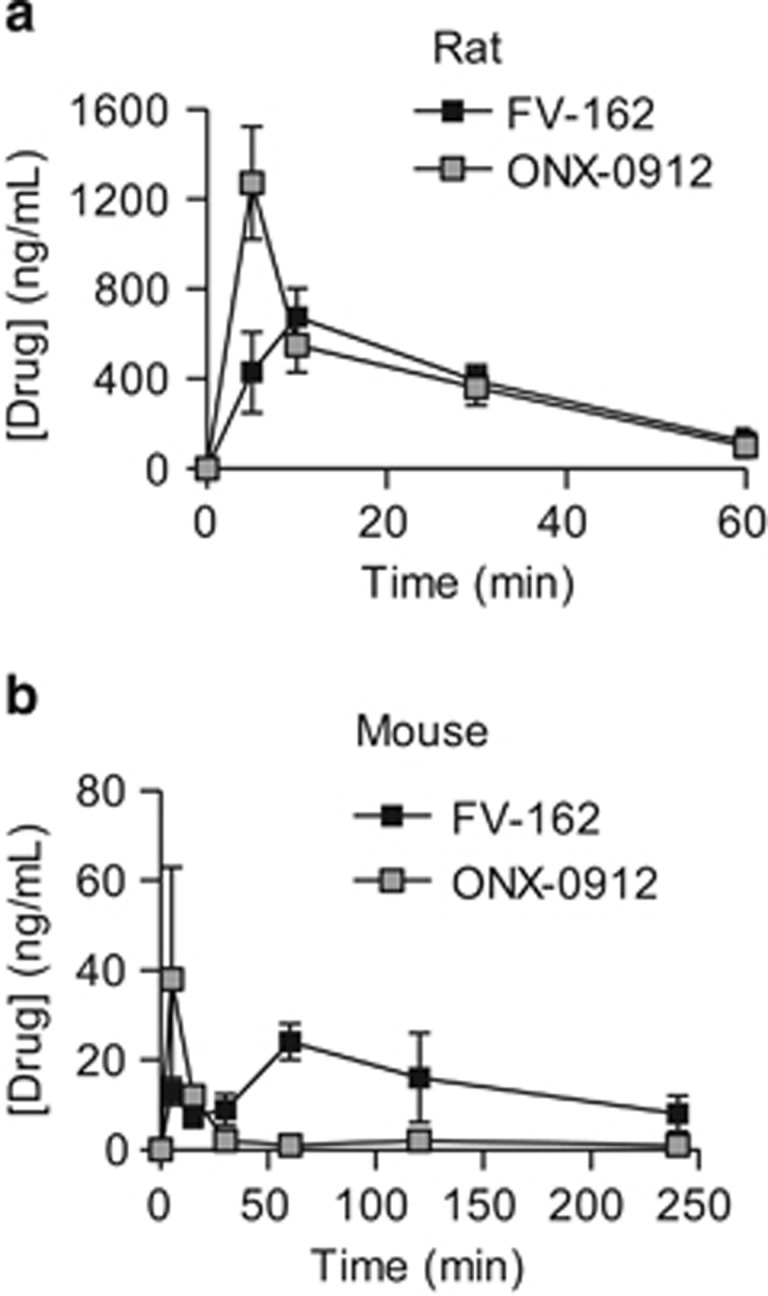
Oral FV-162 displays superior pharmacokinetics compared with ONX-0912. Pharmacokinetics of FV-162 and ONX-0912 in (**a**) rats (40 mg/kg) and (**b**) mice (25 mg/kg) following oral administration. Rodents were administered with FV-162 or ONX-0912, and plasma was collected at increasing times after treatment. Plasma concentration of the two drugs was determined using HPLC. Mean±S.D. plasma levels are shown (*n*=3)

We hypothesized that stabilization of the epoxyketone backbone might improve pharmacokinetic profile and therapeutic index *in vivo* of FV-162. Therefore, we evaluated the pharmacokinetics of FV-162 in comparison with the benchmark inhibitor ONX-0912 in non-obese diabetic/severe combined immunodeficient (NOD/SCID) mice and Sprague-Dawley rats after oral and intravenous administration. Following oral (25 mg/kg) administration in mice, FV-162 demonstrated greater than three-fold higher oral bioavailability and a six-fold increase in drug exposure (AUC; 3117 *versus* 513 min × ng/ml) relative to that of oral ONX-0912. In contrast, FV-162 displayed an almost two-fold decrease in *C*_max_ than that of ONX-0912 (21 *versus* 38 ng/ml) (Figure 4a). Following intravenous (5 mg/kg) administration, FV-162 similarly exhibited more than a four-fold increase in its *t*_1/2_ (95 *versus* 22 min) and a two-fold increase in drug exposure (AUC; 6633 *versus* 3248 min × ng/ml) compared with ONX-0912 (Table 1).

The pharmacokinetic profiles of FV-162 and ONX-0912 following oral administration (40 mg/kg) in rats were similar to mice. FV-162 achieved a greater drug exposure (AUC; 27073 *versus* 22627 min × mg/ml) and a lower *C*_max_ (615 *versus* 1273 mg/ml) compared with ONX-0912 (Figure 4b). Following intravenous (5 mg/kg) administration, FV-162 exhibited an almost seven-fold increase in its *t*_1/2_ (68 *versus* 10 min) and close to a three-fold decrease in *C*_max_ (392 *versus* 1094 ng/ml), relative to ONX-0912 (Table 1).

### Oral FV-162 displays antimyeloma activity in a xenograft model

**Figure 5:**
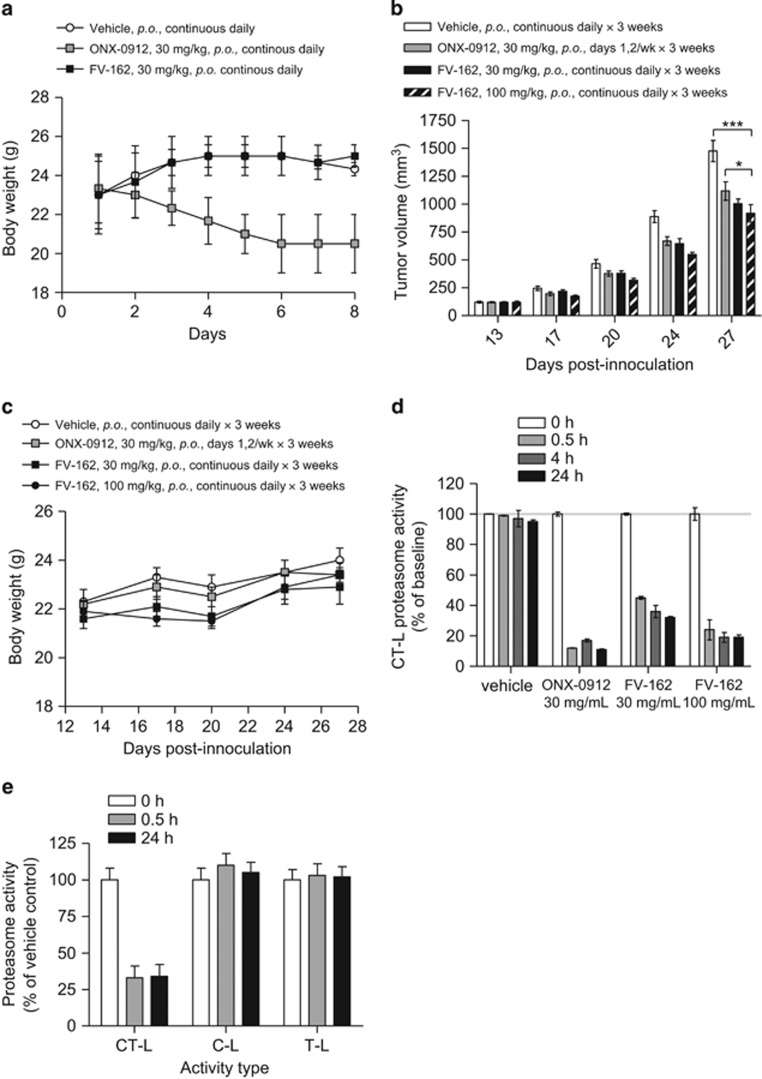
FV-162 displays pharmacodynamic and antimyeloma activity at tolerable doses in mice. (**a**) NOD/SCID mice were treated with FV-162 (30 mg/kg), ONX-0912 (30 mg/kg), or vehicle control (5% DMSO, 20% Cremophor) daily, and body weight was monitored for up to 8 days. Mean±S.E.M., *n*=3. (**b** and **c**) NOD/SCID mice were injected subcutaneously with 5 × 10^6^ cells from the human MM.1 S myeloma cell line. Thirteen days after injection, mice were treated with FV-162 (30, 100 mg/kg by oral gavage), ONX-0912 (30 mg/kg by oral gavage), or vehicle (5% DMSO, 20% Cremophor) on the indicated schedule. Tumor volume (**b**) and body weight (**c**) were monitored over time. Mean±S.E.M., *n*=10. *P<0.05 and ***P<0.001 from a two-way ANOVA with Bonferroni posttests comparing all treatment groups at day 27. (**d**) NOD/SCID mice were treated with ONX-0912 (30 mg/kg by oral gavage), FV-162 (30, 100 mg/kg by oral gavage), or vehicle control (5% DMSO, 20% Cremophor). Lysates were prepared from RBCs at different time points (up to 24 h) after drug treatment, and CT-L proteasome activity was tested. Mean±S.D., *n*=3. (**e**) Lysates prepared from the RBCs at different time points from NON/SCID mice treated with FV-162 (30 mg/ml) were tested for C-L, T-L, and/or CT-L proteasome activity. Mean±S.D., *n*=3

To characterize the preliminary safety profile of FV-162, mice were treated with 30 mg/kg of the drug by oral gavage. Mice treated with FV-162 displayed no evidence of toxicity, behavioral change, or loss of body weight for up to 8 days of treatment. In contrast, daily dosing of ONX-0912 resulted in loss of body weight (Figure 5a). The single maximum tolerated dose (MTD) of FV-162 was 200 mg/kg and that of ONX-0912 was 40 mg/kg by oral gavage in mice.

We next evaluated the safety profile and anticancer efficacy in a subcutaneous xenograft model using the MM.1S human myeloma cells. Mice with established tumors were treated with FV-162 at 30 and 100 mg/kg daily or with ONX-0912 at 30 mg/kg administered twice weekly by oral gavage similar to a previously described schedule for this drug.^20^ All three schedules of proteasome inhibitors significantly reduced tumor burden compared with the vehicle control by 24 days after xenograft inoculation (*P*<0.01), reaching greater statistical significance after 27 days (*P*<0.001). Daily administration of FV-162 at half of its MTD (100 mg/kg) produced a greater reduction in tumor growth by 27 days after xenograft inoculation compared with ONX-0912 at close to its MTD (30 mg/kg) (*P*<0.05; Figure 5b). Tumor reduction after treatment with FV-162 was similar to the effect of carfilzomib (Supplementary Figure S3). All of the regimens tested were well tolerated by the mice, with no substantial effect on mouse body weight observed during treatment (Figure 5c). Thus FV-162 is well tolerated and displays preclinical efficacy with continuous daily dosing with a wide differential activity between malignant and normal cells.

### Oral FV-162 displays pharmacodynamic activity in mice at tolerable doses

We then compared the ability of FV-162 and ONX-0912 to inhibit the CT-L enzymatic activity in isolated red cells after oral administration to mice. Mice were treated with a single dose of oral ONX-0912 (30 mg/kg) or FV-162 (30 and 100 mg/kg). CT-L proteasome activity was measured in red blood cells (RBCs) isolated from the mice up to 24 h postdose (Figure 5d). At equivalent doses (30 mg/kg), FV-162 produced less inhibition of the CT-L activity than ONX-0912. At half the MTD (100 mg/kg), FV-162 performed similarly to almost the MTD of ONX-0912 (30 mg/kg). We also demonstrated that inhibition of proteasome activity in the isolated RBCs was sustained for up to 24 h after treatment with either FV-162 or ONX-0912. In contrast, proteasome activity recovered to >50% of baseline levels by 24 h after treatment with the reversible inhibitor bortezomib (data not shown). In contrast to the effects on the CT-L proteasome inhibition in murine RBCs, administration of FV-162 did not inhibit C-L or on T-L subunit activity in murine RBCs (Figure 5e). Taken together, these data indicate that that FV-162 is an orally bioavailable, selective proteasome inhibitor with a favorable ratio of efficacy to toxicity.

## Discussion

The proteasome inhibitors bortezomib and carfilzomib produce clinical responses in hematological malignancies, such as multiple myeloma and mantle cell lymphoma. Although prolonged proteasome inhibition has been shown to be the most optimal antimyeloma therapy in preclinical models,^16^ toxicity restricts dose intensity of these compounds in the clinical setting. Compared with bortezomib, more selective epoxyketone-based proteasome inhibitors should be capable of prolonged target inhibition by avoiding boronic-acid-based off-target toxicity.^16^ However, first-generation epoxyketone inhibitors such as carfilzomib and ONX-0912 have poor pharmacokinetics, are administered at MTD, and are also associated with severe toxicities. Therefore, selective proteasome inhibitors with optimized pharmacokinetics may achieve similar or even prolonged target inhibition with reduced toxicity. Chemical optimization of this class of therapeutics may lead to an improved therapeutic window.

By testing a panel of fluorinated derivatives of the epoxyketone proteasome inhibitors ONX-0912 and carfilzomib, we identified FV-162 as a novel and orally bioavailable proteasome inhibitor. FV-162 has an improved pharmacokinetic profile that was associated with comparable efficacy and improved safety profile. In studies of rodents, FV-162 had a lower *C*_max_ and higher AUC than ONX-0912 after the administration of an equivalent oral dose. Despite the total greater exposure to the drug, FV-162 was better tolerated in mice xenograft models and displayed a wider differential activity between malignant and normal cells. These results are consistent with previous studies demonstrating that toxicity of irreversible proteasome inhibitors is related more closely to *C*_max_ than the AUC. For example, Yang *et al.*^29^ studied the pharmacokinetics and toxicity of bolus and 30 minute infusions of carfilzomib in rats. At a dose of 8 mg/kg, a bolus infusion of carfilzomib was lethal to 44% of rats, whereas the same dose given over 30 min was not toxic.^29^ Recent human data also support the relationship between the *C*_max_ of epoxyketone inhibitors and toxicity. Patients with multiple myeloma tolerated up to 56 mg/m^2^ carfilzomib without adverse effects when the drug was given as an infusion over 30 min, which is more than double the MTD of carfilzomib when given as a bolus infusion.^30^ Thus increasing the stability and AUC of epoxyketone-based proteasome inhibitors may reduce the need for high instantaneous exposures and concomitant *C*_max_ toxicities.

*In vitro* metabolism studies demonstrated that FV-162 had comparable stability to carfilzomib and greater stability than ONX-0912. Furthermore, a daily dose of FV-162 reduced the tumor volume without a loss in weight in the mouse xenograft model. However, in isolated red blood cells from mice, a single low oral dose of FV-162 produced incomplete inhibition of the proteasome. Taken together, these data suggest that partial inhibition of the proteasome may be sufficient to eradicate malignant cells but spare normal cells. As such, we hypothesize that the proteasome complex operates at near maximal capacity in myeloma and partial inhibition of this complex is cytotoxic to myeloma cells. In contrast, the proteasome complex functions at submaximal capacity in normal cells and has greater reserve to tolerate partial inhibition. Future studies should test this hypothesis by examining the biological capacity of the proteasome complex.

Increased expression of the immunoproteasome subunit *β*5i renders leukemia and myeloma cells more sensitive to proteasome inhibitors, including bortezomib, carfilzomib, and ONX-0912.^31, 32^ Future studies should therefore examine the differential ability of FV-162 and ONX-0912 to inhibit components of the immunoproteasome as well as the constitutive proteasome.

Currently, ONX-0912 is being evaluated in phase 1 and 2 clinical trials, alone and in combination with dexamethasone and sorafenib for hematological malignancies and solid tumors. In a phase 1 study of once-daily orally administered ONX-0912 in patients with advanced refractory or recurrent solid tumors, the MTD was 150 mg/day.^23, 24^ When administered twice daily, the MTD has not been reached at cumulative doses up to 190 mg/day. The most commonly treated side effects were gastrointestinal related and thrombocytopenia.^23^ ^24^ To date, responses have not been reported.

In summary, we have identified a novel proteasome inhibitor FV-162 that has favorable pharmacokinetic and safety profiles for improved dose intensity. Continuous daily dosing of FV-162 well below its MTD appears more optimal for exploiting the proteasome dependency of malignant cells while minimizing toxicities. Thus, given the advantages of FV-162 observed in this study, clinical investigation of this promising agent is warranted.

## Materials and Methods

### Compounds and reagents

All proteasome inhibitors except bortezomib were synthesized and provided by Fluorinov Pharma, Inc. (Toronto, ON, Canada). Bortezomib was purchased from Millennium Pharmaceuticals, Inc. (Cambridge, MA, USA). Fluorogenic proteasome substrates were obtained from Enzo Life Sciences (Plymouth Meeting, MA, USA). Compounds and proteasome substrates were dissolved in dimethyl sulfoxide (DMSO) to stock concentrations of 10 and 2 mM, respectively, and stored at −20 °C.

### Synthesis of FV-162

FV-162 was synthesized by initially coupling fluorinated serine derivatives 1 and 2 (Figure 1) by using HBTU to give a boc/benzyl-protected dipeptide, 3. This was hydrogenated to remove the benzyl group and the resulting carboxylic acid was coupled with an epoxyketone derivative of phenylalanine to give compound 5. The boc-protecting group was then cleaved using TFA and DCM giving compound 6 as a trifluoroacetate salt, which was, in turn, coupled with 2-methylthiazole-5-carboxylic acid (using HBTU) to give the desired product, FV-162.

### Cells and cell culture

Human multiple myeloma cell lines 8226, H929, JJN3, KMH11, KMS11, KMS18, LP1, MM.1 S, OPM2, and U266 were grown in Iscove's modified Dulbecco's medium (IMDM). Cell lines were authenticated with short tandem repeat method in September 2011. In addition, cell lines were continually monitored by morphological inspection. Primary mononuclear cells were isolated from peripheral blood or bone marrow samples from multiple myeloma patients at the Princess Margaret Cancer Centre (Toronto, ON, Canada) by Ficoll density gradient centrifugation and cultured in IMDM. All cell culture media were obtained from the Ontario Cancer Institute Tissue Culture Media Facility (Toronto, ON, Canada) and were supplemented with 10% fetal calf serum, 100 *μ*g/ml penicillin, and 100 *μ*g/ml streptomycin (Hyclone, Logan, UT, USA). All cells were grown in a humidified incubator at 37 °C with 5% CO_2_.

### Metabolic stability assay

Pooled liver microsomes isolated from male CD-1 mice were purchased from BD Biosciences (San Jose, CA, USA). Metabolic stability was calculated by measuring the percentage of 5 *μ*M of each test compound remaining after 15 min incubation with 0.5 mg/ml liver microsomes at 37 °C. Compounds were detected by LC/MS using a Xevo quadrupole time-of-flight mass spectrometer and an ACQUITY ultra-performance LC (UPLC) system (Waters Inc., Milford, MA, USA) to determine relative peak areas of each parent compound.

### Cell viability assays

Cellular viability was primarily assessed by MTS assay according to the manufacturer's instructions (Promega, Madison, WI, USA). Ten thousand cells per well were seeded in triplicate in tissue culture-treated 96-well plates. Two hours after seeding, cells were treated with proteasome inhibitors for 72 h (0.01–10 *μ*M) or DMSO vehicle control. Cell viability was independently confirmed by reading the optical density at 490 nm and by exclusion of trypan blue stain (Invitrogen, Burlington, ON, Canada). Viability of primary mononuclear cells was determined by Annexin V–fluorescein isothiocyanate and propidium iodide co-staining (Biovision Research Products, Mountain View, CA, USA) using flow cytometry according to the manufacturer's instructions after 48 h of treatment with inhibitors or vehicle control.

### Proteasome activity assays

#### Cell lysates

Cells were harvested by centrifugation at 1200 r.p.m. at room temperature. Cell pellets were washed with PBS and lysed with assay lysis buffer (50 mM HEPES (2-[4-(2-hydroxyethyl) piperazin-1-yl]ethanesulfonic acid)), pH 7.5; 150 mM NaCl; 1% Triton X-100; 2 mM ATP). Cell lysates were incubated on ice for 30 min, mixed by vortex every 5 min, and centrifuged at 12 000 × g for 10 min. Supernatants were transferred to a 96-well plate in triplicate. For each assay, 10 *μ*g of total protein were incubated for 1 h at 37 °C with increasing concentrations (0.001–10 *μ*M) of test compound or vehicle control diluted in assay buffer (50 mM Tris-HCl (tris(hydroxymethyl)aminomethane-HCl), pH 7.5; 150 mM NaCl). After incubation, a specific fluorogenic proteasome substrate was added to each assay reaction (40 *μ*M in a total volume of 100 *μ*l). N-Succinyl-Leu-Leu-Val-Tyr-7-amino-4-methylcoumarin (Suc-Leu-Leu-Val-Tyr-AMC) was used for measuring CT-L activity, t-butoxycarbonyl- Leu-Arg-Arg-7-amino-4-methylcoumarin (Boc-Leu-Arg-Arg-AMC) for T-L activity, and benzyloxycarbonyl-L-leucyl-L-leucyl-L-glutamyl-7-amino-4-methylcoumarin (Z-Leu-Leu-Glu-AMC) for C-L activity. The excitation wavelength was 360 nm and the fluorescence emission of free AMC released during the enzymatic reaction was detected at 460 nm on a SpectraMax M5 fluorescent spectrophotometric plate reader (Molecular Devices, Sunnyvale, CA, USA). AMC release rate was measured at 37 °C in a kinetic mode, recording every 5 min for 30 min.

#### Murine red blood cells

Five-to-six-week-old male NOD/SCID mice were administered vehicle (5% DMSO, 20% Cremophor) or proteasome inhibitors either intravenously or by oral gavage (3 mice per group), and 20–50 *μ*l of venous blood were collected from each mouse over 24 h. Blood samples were mixed with heparin (APP Pharmaceuticals; Schaumburg, IL, USA) in 0.5-ml tubes in accordance with the manufacturer's instructions. After centrifugal separation at 3000 × g for 10 min, RBCs in the bottom layer were transferred into a new tube and stored at −70 °C until use. RBCs were lysed with assay lysis buffer and incubated on ice for 30 min, mixing by vortex every 5 min, and centrifuged at 12 000 × g for 10 min. Supernatants were transferred to a 96-well plate and proteasome activity was measured as described in 'Cell lysates' section.

### Dose tolerance studies

NOD/SCID mice were administered FV-162 (30 mg/kg daily, 7 days/week) in a 5% DMSO, 20% Cremophor vehicle by oral gavage or ONX-0912 (30 mg/kg daily, 7 days/week) in a 5% DMSO, 20% Cremophor vehicle by oral gavage for a week. Mice were monitored for changes in body weight, behavior, and physical appearance.

### Pharmacokinetic studies

NOD/SCID mice were administered FV-162 or ONX-0912 at doses of 5 mg/kg intravenously or 25 mg/kg by oral gavage in a 12.5% ethanol, 12.5% Tween-80 vehicle (3 mice per group). Venous blood samples (20–50 *μ*l) were collected at 5, 15, 30 min, and 1, 2, and 4 h postdose. Plasma was separated from whole blood by centrifugation and stored at −20^o^C until analysis. Male Sprague-Dawley rats were administered FV-162 or ONX-0912 at doses of 5 mg/kg intravenously or 40 mg/kg by oral gavage in a 12.5% ethanol, 12.5% Tween-80 vehicle (3 rats per group). Venous blood samples (20–50 *μ*l) were collected at 5, 10, 30, and 60 min postdose. Plasma was separated from whole blood by centrifugation and stored at −20 ^o^C until analysis. The *C*_max_, its associated time (*T*_max_), the terminal half-life (*t*_1/2_), area under the plasma concentration–time curve (AUC), clearance (CL), and volume of distribution (*V*_d_) were evaluated using Pheonix WinNonlin 6.2.1 (Princeton, NJ, USA), and relative oral bioavailability (*F*%) values were calculated as follows: *F*%=AUC_oral gavage_/AUC_intravenous_ × 100.

### Efficacy in xenograft model

Human MM.1S myeloma cells (5 × 10^6^) were injected subcutaneously into 6–8-week-old female NOD/SCID mice (10 mice per group). When tumors were palpable, mice were administered vehicle by oral gavage (5% DMSO, 20% Cremophor; once daily × 3 weeks), carfilzomib intravenously (5 mg/kg, once daily on days 1 and 2 of each week × 3 weeks), ONX-0912 by oral gavage (30 mg/kg, once daily on days 1 and 2 of each week × 3 weeks), or FV-162 by oral gavage (30 mg/kg or 100 mg/kg, once daily × 3 weeks). Over time, tumor volume (tumor length × width^2^ × 0.5236) was measured with calipers (Control Company, Friendswood, TX, USA), and mouse body weight was monitored.

### Statistics

Unless otherwise indicated, all experiments have been performed independently at least three times. Half-maximal inhibitory concentration (IC_50_) values were calculated using Sigmaplot 8.0 (SPSS Inc., Chicago, IL, USA), computing a four-parameter logistic equation from inhibition curves. Total drug exposure in mice and *C*_max_ in rats was compared using an unpaired *t*-test. Inhibitor efficacy in the myeloma xenograft model was compared using a two-way ANOVA with Bonferroni posttests comparing all treatment conditions.

### Study approvals

All mouse experiments were performed in accordance with approval from the Ontario Cancer Institute Animal Care Committee or the approval for the CRO. The collection and use of human tissue was approved by the University Health Network Research Ethics Board, and samples were obtained with informed consent.

## Figures and Tables

**Table 1:** Pharmacokinetics of FV-162 and ONX-0912 in mice and rats following intravenous or oral administration

				
**Pharmacokinetic parameter**			**FV-162**		**ONX-0912**	
	**5 mg/kg i.v.**	**25 mg/kg p.o.**	**5 mg/kg i.v.**	**25 mg/kg p.o.**	
*Mouse*				
*C*_max_, ng/ml	268	21	187	38
*T*_max_, min	5	60	5	5
AUC, min × ng/ml	6633	3117	3248	513
*t*_1/2_, min	95	*ND*	22	*ND*
CL, ml/min/kg	702	*ND*	1489	*ND*
*V*_d_, l/kg	96	*ND*	48	*ND*
F, %	NA	10	NA	3

**Pharmacokinetic parameter**			**FV-162**		**ONX-0912**	
	**5 mg/kg i.v.**	**40 mg/kg p.o.**	**5 mg/kg i.v.**	**40 mg/kg p.o.**	
*Rat*				
* C*_max_, ng/ml	392	615	1094	1273
* T*_max_, min	5	15	5	5
AUC, min × ng/ml	24 184	27 073	18 070	22 627
* t*_1/2_, min	68	ND	10	ND
CL, ml/min/kg	1345	ND	273	ND
*V*_d_, l/kg	20	ND	4	ND
*F*, %	NA	14	NA	16

Abbreviations: i.v., intravenous; NA, not applicable; ND, not determined; p.o., oral

## Abbreviations

C-L: caspase-like
T-L: trypsin-like
CT-L: chymotrypsin-like
NOD/SCID: nonobese diabetic/severe combined immunodeficiency
RBC: red blood cell
MTD: maximum tolerated dose
AUC: area under the curve
*C*_max_: peak plasma concentration
*T*_max_: time associated with *C*_max_
*t*_1/2_: terminal half-life
CL: clearance
*V*_d_: volume of distribution
