# Supplementary material for: FV-162 is a novel, orally bioavailable, irreversible proteasome inhibitor with improved pharmacokinetics displaying preclinical efficacy with continuous daily dosing
Source: Cell Death Dis. 2015 Jul 9;6(7):e1815–. doi: 10.1038/cddis.2015.187 (PMC4650734; doi:10.1038/cddis.2015.187)
Supplement: Supplementary Information [file cddis2015187x1.pdf]

FV-162 is a novel, orally bioavailable, irreversible proteasome inhibitor with improved pharmacokinetics that displays preclinical efficacy with continuous daily dosing

Zezhou Wang,[1,2] Peter Dove,[2] Xiaoming Wang,[1] Aisha Shamas-Din,[1] Zhihua Li,[1] Alex Nachman,[1] Yoonsun Jenny Oh,[1] Rose Hurren,[1] Amy Ruschak,[3] Shane Climie,[2] Barry Press,[4] Carly Griffin,[4] Elijus Undzys,[4] Ahmed Aman,[4] Rima Al-awar,[4] Lewis Kay,[3] David O'Neill[2,4], Suzanne Trudel,[1] Malik Slassi,[2] and Aaron D. Schimmer[1]

[1]Princess Margaret Cancer Centre, Toronto, ON, Canada; [2]Fluorino v Pharma Inc., Toronto, ON, Canada; [3]Department of Biochemistry, University of Toronto, Toronto, ON, Canada; and [4]Ontario Institute for Cancer Research, Toronto, ON, Canada

Running Title: FV-162, a novel oral proteasome inhibitor

Supplemental Figure Legends

Supplemental Figure S1. Novel analogs of the epoxyketone proteasome inhibitors carfilzomib and ONX-0912. Fourteen novel structural analogs of the irreversible epoxyketone proteasome inhibitors carfilzomib (tetrapeptide, administered intravenously) and ONX-0912 (tripeptide, orally bioavailable) were synthesized in this study using a novel fluorine-based chemistry technology (Fluorinov Pharma Inc., Toronto, ON, Canada).

Supplemental Figure S2. Identification of FV-162 as the lead proteasome inhibitor compound with potent antimyeloma activity. The novel proteasome inhibitor analogs were characterized in comparison to the benchmark proteasome inhibitors bortezomib, carfilzomib, and/or ONX-0912 (black bars). Horizontal grey lines delineate the range of values encompassed by these benchmark compounds, and novel analogs with equivalent or improved activity or stability are highlighted in grey bars (others in white). The novel analog FV-162 is highlighted in each panel by an arrow. (**A**) The metabolic stability of compound shown was determined in the presence of pooled mouse liver microsomes. The percentage of 5 μM of each test compound remaining after a 15 minute incubation with 0.5 mg/mL liver microsomes was detected by liquid chromatography/mass spectroscopy (LC/MS). Antipyrine and testosterone (5 μM each) were included as positive and negative controls, respectively (hatched bars). (**B**) Chymotrypsin-like (CT-L) proteasome activity present in KMS11 whole cell lysates was determined through specific cleavage of a fluorogenic substrate following exposure to each compound for 2 hours. Sensitivity was evaluated using the IC_{50}. (**C**) Cell growth and viability of the human myeloma cell line KMS11 was assessed using the MTS assay after exposure to

each compound for 72 hours. Sensitivity was evaluated using the half-maximal inhibitory concentration (IC_{50}).

Supplemental Figure S3. FV-162 displays similar antimyeloma activity to carfilzomib. NOD/SCID mice were injected subcutaneously with 5 x 106 cells from the human MM.1S myeloma cell line and treated with FV-162, carfilzomib, or vehicle (5% DMSO, 20% Cremophor) as indicated. Tumor volume was monitored over time. Data represent the mean ± s.e.m. from 10 mice per group.

carfilzomib

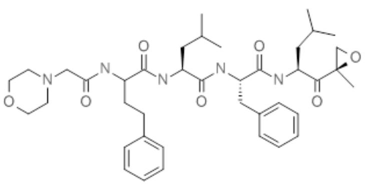

ONX-0912

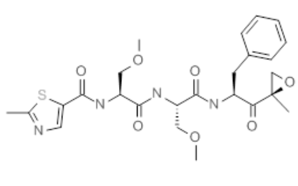

FV-162

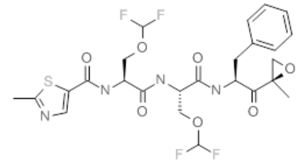

FV-208

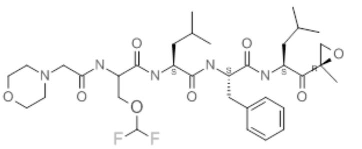

FV-206

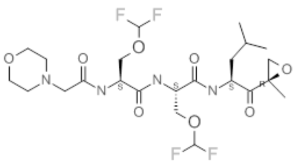

FV-212

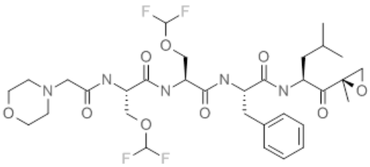

FV-209

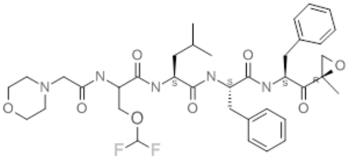

FV-201

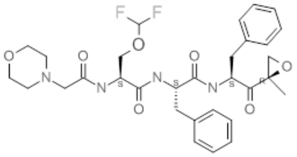

FV-204

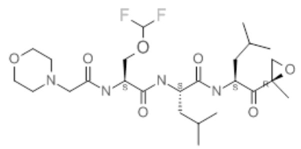

FV-210

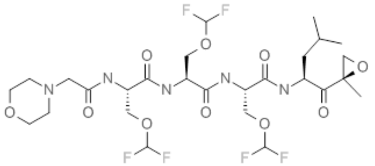

FV-202

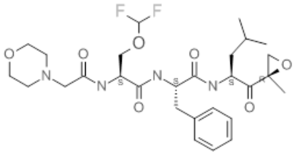

FV-213

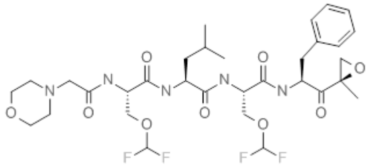

FV-211

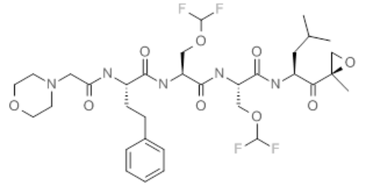

FV-203

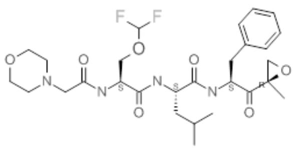

FV-205

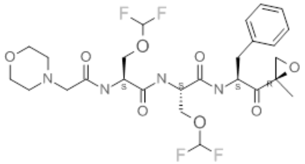

FV-214

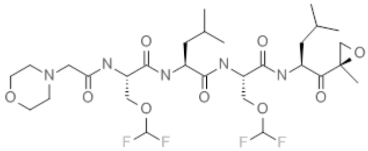

Supplementary Figure 1

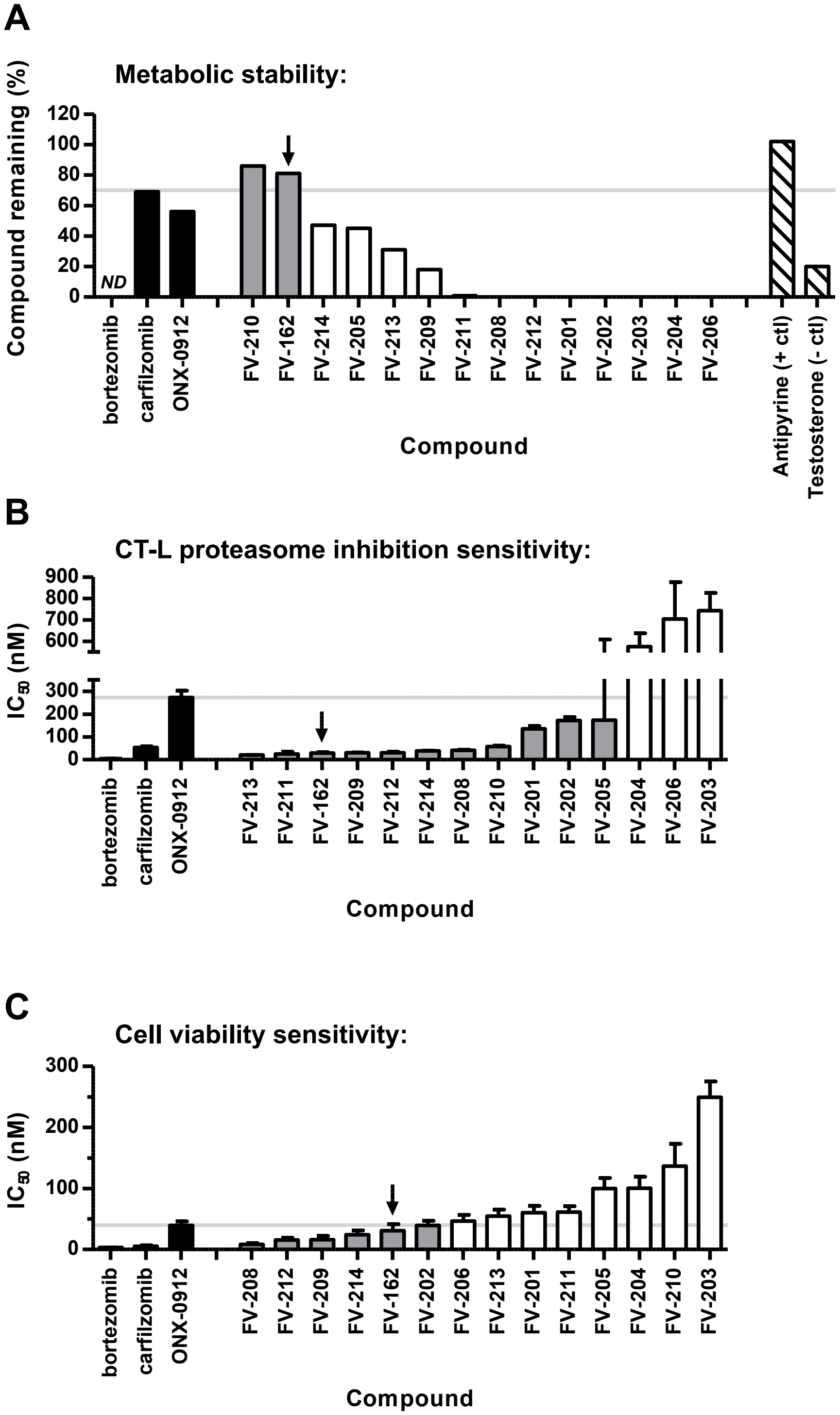

Supplementary Figure 2

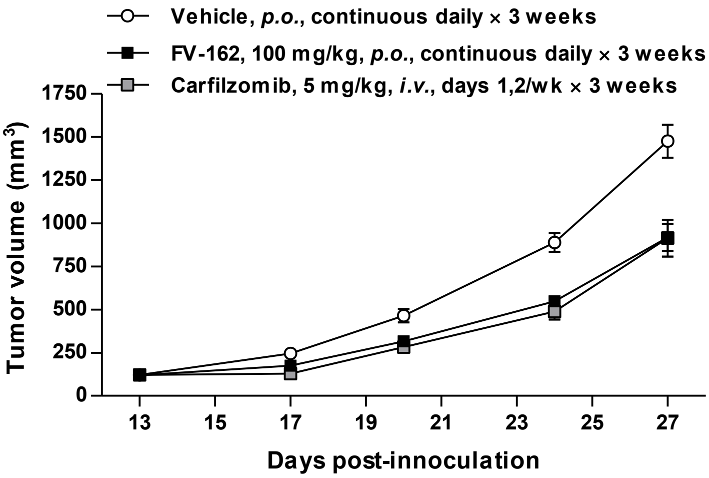

Supplementary Figure 3
